# Experimental Investigation on the Effect of Surface Shape and Orientation in Magnetic Field Assisted Mass Polishing

**DOI:** 10.3390/mi13071060

**Published:** 2022-06-30

**Authors:** Yee-Man Loh, Chi-Fai Cheung, Chunjin Wang, Lai-Ting Ho

**Affiliations:** State Key Laboratory in Ultra-Precision Machining Technology, Department of Industrial and Systems Engineering, The Hong Kong Polytechnic University, Hong Kong, China; yee-man-kristy.loh@connect.polyu.hk (Y.-M.L.); lai.ting.ho@polyu.edu.hk (L.-T.H.)

**Keywords:** magnetic field assisted finishing, polishing, material removal, surface shape, ultra-precision machining

## Abstract

Magnetic field assisted finishing (MFAF) technology has been widely used in industries such as aerospace, biomedical, and the optical field for both external and internal surface finishing due to its high conformability to complex surfaces and nanometric surface finishing. However, most of the MFAF methods only allow polishing piece-by-piece, leading to high post-processing costs and long processing times with the increasing demand for high precision products. Hence, a magnetic field-assisted mass polishing (MAMP) method was recently proposed, and an experimental investigation on the effect of surface posture is presented in this paper. Two groups of experiments were conducted with different workpiece shapes, including the square bar and roller bar, to examine the effect of surface orientation and polishing performance on different regions. A simulation of magnetic field distribution and computational fluid dynamics was also performed to support the results. Experimental results show that areas near the chamber wall experience better polishing performance, and the surface parallel or inclined to polishing direction generally allows better shearing and thus higher polishing efficiency. Both types of workpieces show notable polishing performance where an 80% surface roughness improvement was achieved after 20-min of rough polishing and 20-min of fine polishing reaching approximately 20 nm.

## 1. Introduction

In most of the machining processes for ultra-smooth surfaces applied in various fields such as optics, imaging, biomedical engineering, and the aerospace and automotive fields [1,2], polishing is usually required as a final and crucial step to remove defects and to smoothen the surfaces. Different kinds of polishing technologies have been developed, including ion beam finishing [3], bonnet polishing [4], fluid jet polishing [5], plasma finishing [6], magnetic field assisted finishing (MFAF) [7], etc. MFAF technology, being one of the most promising technologies in achieving ultra-smooth surface finishing, was first developed in the 1930s and has been widely researched and applied in various industries such as aerospace, biomedical, optics, etc. [7,8,9]. This technology shows advantages over polishing difficult-to-access surface [10], freeform surface [11] and microstructures [12] due to its high flexibility and conformity to the workpiece shape. MFAF can now be divided into two main streams depending on the polishing media: Magnetic Abrasive Finishing (MAF) and Magnetorheological Finishing (MRF).

MAF adopts magnetic abrasive particles which are made up of the combination of magnetic particles and abrasive particles to perform surface finishing [13]; with a different combination of magnetic abrasive particles and magnetic poles movement, both external and internal surface finishing can be achieved. Guo et al. [14] developed a localized vibration-assisted magnetic abrasive polishing method for various types of microstructures and achieved surface roughness reduction by 80% while maintaining the form accuracy. A rotating-vibrating MAF process was also proposed [15] for additive manufactured components with complex internal structures, which successfully improved surface roughness from 7 μm to 0.5 μm on both surfaces within 3 h. Vahdati and Rasouli [11] studied and optimized the parameters for freeform surface MAF polishing and reported the characteristic of magnetic abrasives. Sumit and Chhikara [16] and Deepak et al. [17] proved that MAF is able to efficiently polish flat surfaces, internal and external surfaces of tube-like workpieces to a mirror surface with surface roughness on the order of a few nanometers. Amnieh et al. [18] presented a MAF setup which is able to finish internal grooves of a cylindrical tube, and various experiments on the effectiveness of parameters were performed. With the trapezium shaped grooves, a permanent magnet tool was ground to form the appropriate shape to ensure uniform gap distance between the magnetic tool and workpiece wall. Experimental results indicate that the proposed MAF setup is able to improve the surface quality of internal grooves by 70%, from 1.12 μm to 0.32 μm, with only 30 mg material loss, which further verifies the capability of MAF in internal surface finishing. Mulik and Pandey [19] developed the ultrasonic-assisted magnetic abrasive finishing (UMAF) process which combines ultrasonic vibration and MAF to achieve quick surface finishing, and was successfully achieved to polish a hardened steel workpiece to 22 nm in 80 s. Sihag et al. [20] further enhanced the UMAF process into a chemo ultrasonic assisted magnetic abrasive finishing, resulting 86% surface roughness reduction. Research on magnetic abrasive particles (MAPs) was also conducted in the literature [21,22], which summarized the polishing performance of various types of MAPs fabricated by different processes, and concluded that sintered MAPs show the strongest bonding and highest material removal.

On the other hand, MRF utilizes a magnetorheological fluid which stiffens under the magnetic field to create a highly conforming abrasive lap to polish the workpiece surface [23], in which the stiffness and shape of the formed MR brush can be controlled by the magnetic field strength [24]. This technology has been widely used and commercialized in the field of high-precision surface finishing, which is capable of achieving the nanometric finishing of freeform optics. MRF was first invented by Kordonski et al. in the 1980s, and the first computer-controlled MRF machine prototype was proposed later in 1995 [25,26]. Kordonski et al. [27] then widened the application of MRF to freeform and concave surfaces by introducing Magnetorheological Jet Finishing (MRJF), which is able to provide a more stable and precise material removal function compared to the common abrasive-water jet polishing. Experimental results proved that MRJF could produce ultra-precise surfaces on the order of tens of nanometers peak-to-valley together with surface roughness smaller than 1 nm rms on various materials such as metals, ceramics and glasses. Pattanaik and Agarwal [28] and Kumar et al. [29] innovatively developed a different MRF process of Rotational-Magnetorheological abrasive flow finishing (R-MRAFF), and it was pointed out that the magnetic flux density and the polishing angle (inclination) of the polished surface were two significant parameters affecting the roughness improvement and uniformity. A steel knee joint implant of 200 nm initial roughness was polished to a mirror surface, which has verified its practicality in the biomedical field. Saraswathamma et al. [30] conducted experiments on the ball end MRF to investigate the effects of parameters on silicon wafers and revealed that the working gap plays a crucial role in surface roughness improvement. Kansal et al. [31] introduced an innovative tool for MRF of diamagnetic materials which is capable of reducing surface roughness from 274 nm to 29 nm within 7.5 min, further widening its application in the electronics field. Anwesa and Manas [32] developed a polishing tool for freeform surface finishing and adopted finite element analysis to determine the optimal design configuration of the tool; experiments were conducted on a titanium workpiece and 95% surface roughness improvement was obtained from 180 nm to 10 nm. An MRF method for internal surface finishing of titanium tubes was also proposed and the effect of various parameters were investigated and optimized [33]. The application of MRF in polishing alloys and ceramics was also investigated and was proven to have high efficiency in achieving nanometer scale surface roughness without surface or subsurface damage [34,35,36].

However, most of the above MFAF processes focus on precision polishing of the workpiece one-by-one, which leads to high polishing costs and is time consuming when polishing a large amount of workpieces. With the increasing demand of ultra-precision complex and freeform surfaces, more and more attention has been paid to increase the production efficiency of the polishing process. Hence, our research group [37] recently developed a novel magnetic field assisted mass polishing (MAMP) system which can polish tens of freeform surfaces simultaneously and obtain nanometric surface roughness. A feasibility study and the effects of some key parameters have been conducted and measured in our previous research. However, the target surface for polishing in our previous research [38,39] was only one side surface facing the external wall of the annular chamber.

Hence, an experimental investigation on the effects of the workpiece posture and orientation during MAMP was conducted in this paper. The experimental setup and design of experiments are presented in Section 2. Section 3 and Section 4 present the experimental results of the polishing experiments as well as the discussion on the simulated computational fluid dynamics (CFD) model and magnetic field simulation. Finally, a conclusion is presented to summarize the research work in this paper in Section 5.

## 2. Experimental Procedures

### 2.1. Experimental Setup

**Figure 1:**
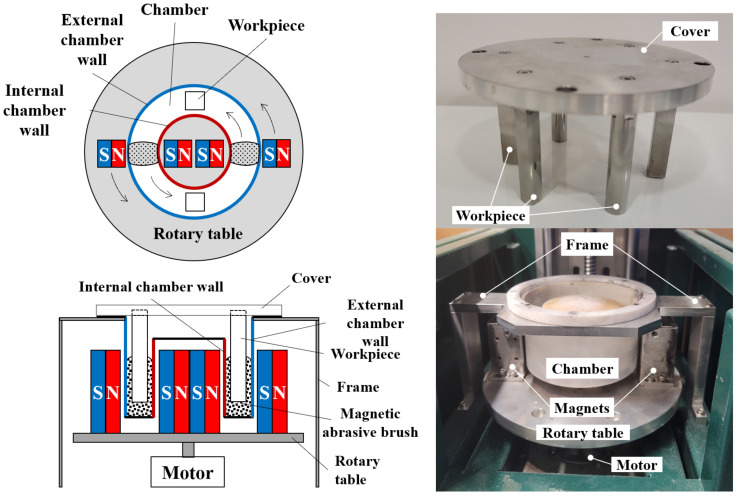
Schematic diagram of the MAMP device.

The schematic diagram of the MAMP device is shown in Figure 1. In this device, workpieces are mounted on the cover and inserted into a Teflon (PTFE) annular chamber for polishing. The chamber is held by a frame to reduce vibration during polishing. Magnetic abrasives are poured into the chamber and two permanent magnetic pairs are placed around the chamber. Magnetic abrasives were then attracted towards the magnetic poles, forming two magnetic abrasive brushes within the chamber. The magnets were controlled to rotate along the fixed annular chamber, driving the abrasive brushes to continuously impinge on the workpiece surface leading to material removal.

In this experiment, bonded magnetic abrasives which are made of iron particles (i.e., average 100~200 µm, 80 wt.%) and alumina abrasive (i.e., average ~2 µm, 20 wt.%), lubricated by silicon oil were used for rough polishing of the workpieces [39], while loose magnetic abrasive composed of carbonyl iron particles (CIP) (i.e., average ~3 µm, 80 wt.%) and polishing fluid (i.e., 150 nm alumina mixed with carrier fluid, 20 wt.%) were used for fine polishing of workpiece [38].

**Figure 2:**
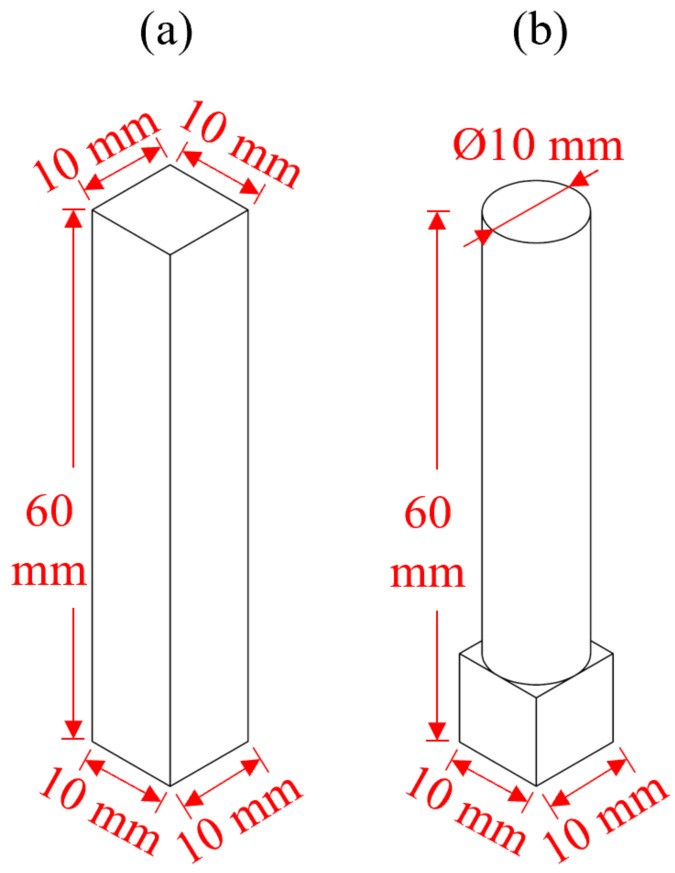
Workpiece design (**a**) square bar (**b**) roller.

Two different shapes of workpiece were prepared as shown in Figure 2, including the square bar and roller. The side length of the square bar is 10 mm, and the diameter of the roller is also 10 mm. The material is 304 stainless steels (SS304). All workpieces were lapped with #400 silicon carbide (SiC) sandpaper before polishing.

### 2.2. Experimental Design

**Figure 3:**
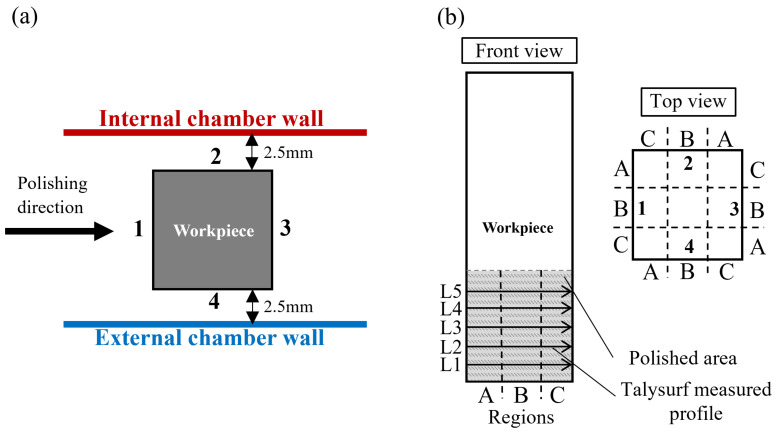
Square bar workpiece surface roughness measurement illustration (**a**) planes (**b**) regions.

To further investigate the polishing performance of the MAMP device, two groups of experiments were designed. In each experiment, the workpieces were first rough polished for 20 min and then fine polished for another 20 min with the polishing parameters as presented in Table 1. The first group of experiments was conducted on rectangular bars to evaluate the polishing performance on flat surfaces and examine the effect of surface orientation. Two square bar workpieces were fixed on the chamber cover and mounted into the chamber, with a 2.5 mm gap distance between both the external and internal wall of the chamber. The polishing performance was evaluated based on the surface roughness of all four surfaces of the workpiece, namely plane 1~4 as shown in Figure 3. Plane 1 and plane 3 were perpendicular to the polishing direction, while plane 2 and plane 4 were parallel to the polishing direction. Each plane was divided into three regions (A, B, C) for surface roughness analysis. The workpiece was measured every 5 min. A Taylor Hobson Talysurf profilometer PGI1240 was used to measure the arithmetic surface roughness ($S_{a}$) of all four surfaces of the workpiece, and five measurements with a total length of 9 mm were taken on each surface, and a Gaussian filter and 0.08 mm cutoff length ($L_{c}$) was applied for surface roughness analysis. The surface form profile was also measured by PGI1240 profilometer.

**Figure 4:**
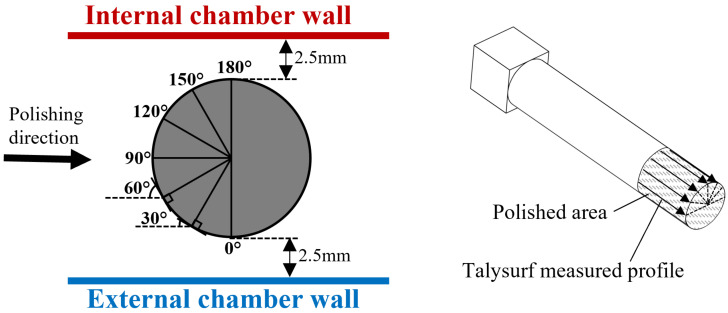
Roller workpiece surface roughness measurement illustration.

The second group of experiments was aimed at studying the polishing performance of an MAMP device on the roller surface and to observe the effect of different impact angles. Two roller workpieces were rough polished for 20 min and then fine polished for another 20 min; seven measurement profiles were taken vertically along the workpiece as shown in Figure 4, including 0°, 30°, 60°, 90°, 120°, 150° and 180°. Theoretically, the polishing direction would form different angles with the tangents of a circle at different positions, which implies a different impingement angle of the abrasive brush on the workpiece surface. Measurements were taken along each angle of the workpiece every 5 min of polishing to record the surface roughness change at each angle.

## 3. Results

### 3.1. Polishing Performance in Four Different Orientations

**Figure 5:**
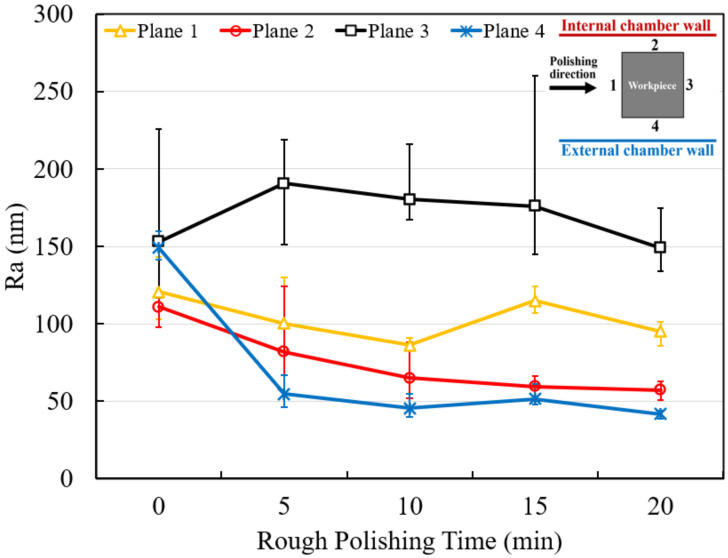
Overall surface roughness of rough polished square bar.

Figure 5 shows the surface roughness results measured in each plane of the square bar workpiece after 20 min of rough polishing. The result shows that plane 1, which was perpendicular to the polishing direction, has very little improvement, whereas plane 2 and plane 4, which were parallel to the polishing direction, shows a significant roughness reduction. The lowest surface roughness was obtained in plane 4, and a drastic decrease can be observed in the first 5 min. Moreover, plane 4 has achieved the limits of rough polishing of the MAMP system at around 40–50 nm, which is around 70% surface roughness convergence. A significant decrease in surface roughness deviation can be observed in plane 1, 2 and 4 after 20 min polishing, however, plane 3 shows no improvement in surface roughness after rough polishing. The reason is that plane 3 was not able to be polished in this case. Under an anticlockwise polishing direction, the surface behind the workpiece (plane 3) can hardly be polished, as the magnetic abrasive brush cannot make contact with the surface under a high-speed rotational movement.

**Figure 6:**
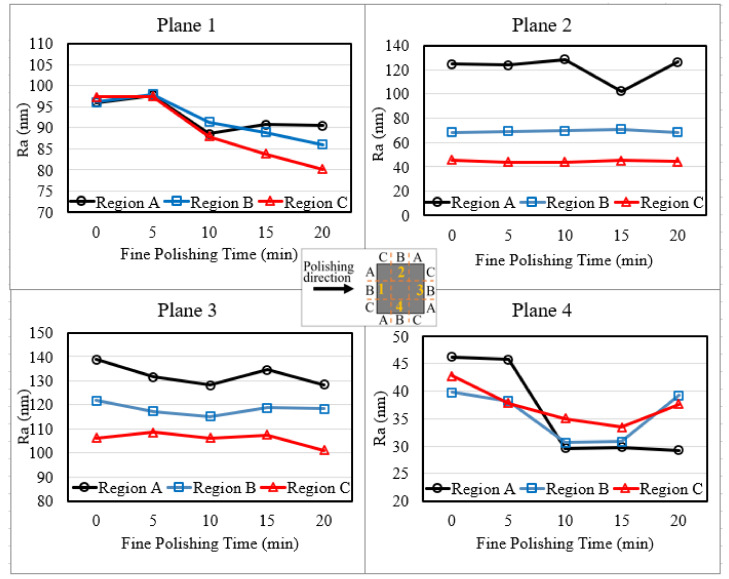
Planer surface roughness of fine polished square bar workpiece.

The rough polished workpieces were then fine polished for 20 min and the results are shown in Figure 6. The surface roughness of plane 2 before fine polishing shows 3 different starting points varying from 40 to 120 nm, indicating an uneven polishing performance on the three regions of plane 2 during rough polishing. It reveals that region A experienced the least material removal; region B was slightly polished while region C was fully polished, reaching around 40 nm roughness.

**Figure 7:**
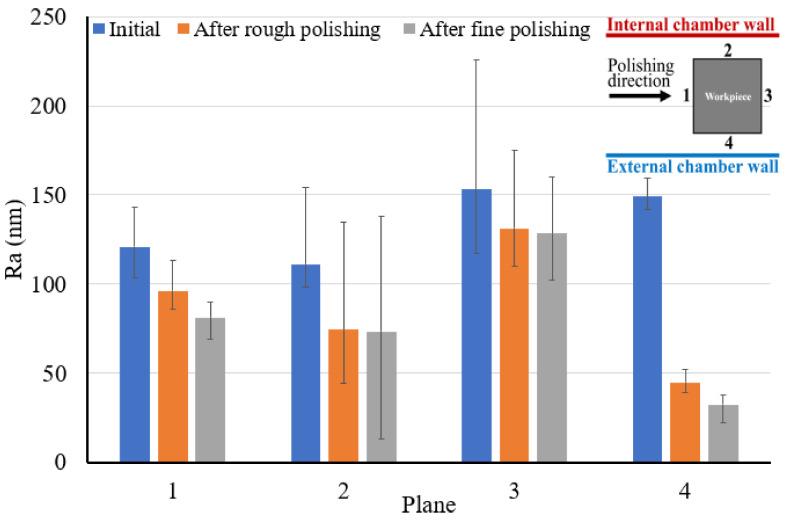
Summary of average surface roughness in each plane of square bar.

Looking into the fine polishing results, it can be seen that all regions in plane 2 and 3 show nearly no improvement along the 20 min fine polishing, and only plane 4 shows a significant decrease in surface roughness to around 30 nm, with region A having the lowest value. Figure 7 has summarized the average surface roughness of each plane before polishing, after rough polishing and after fine polishing. It can be observed that plane 4 shows the most significant improvement, and plane 2 has recorded the largest surface roughness deviation within the plane after fine polishing.

**Figure 8:**
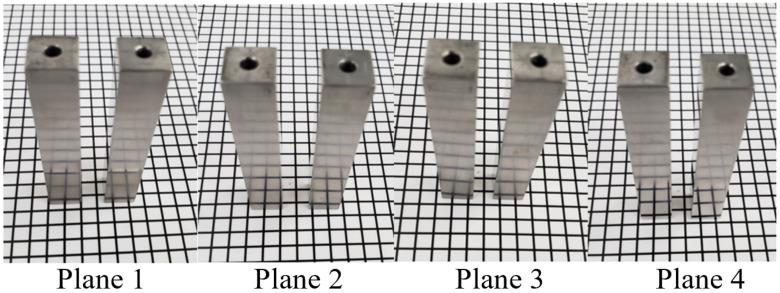
Snapshots of square bar after fine polishing.

**Figure 9:**
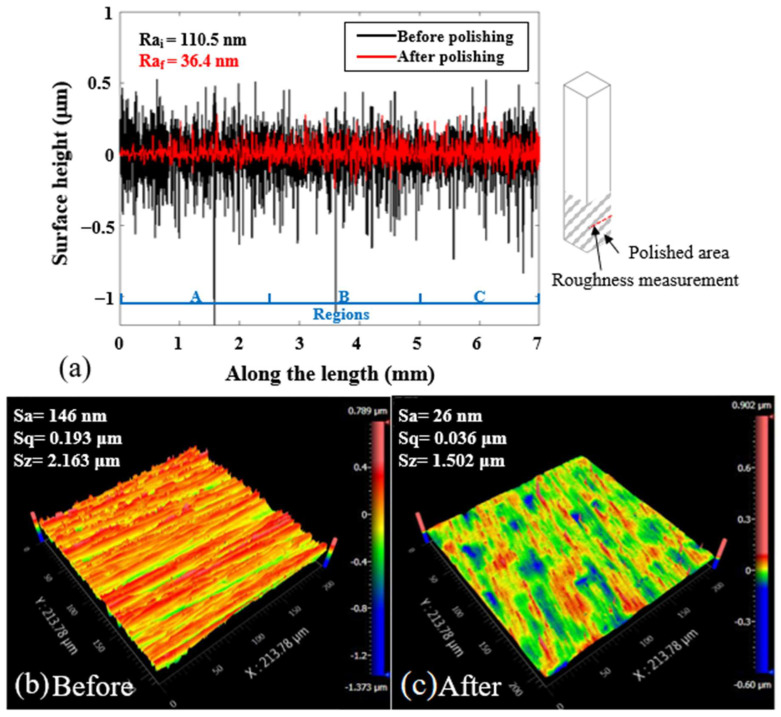
Surface roughness (**a**) profile of square bar plane 4 (L3); Ra_i_ signifies the arithmetic roughness of the profile before polishing. Ra_f_ signifies the arithmetic roughness of the profile after fine polishing. 3D surface topography of the square bar plane 4 (**b**) before and (**c**) after polishing, measured from a ZYGO Nexview 3D optical interferometer.

**Figure 10:**
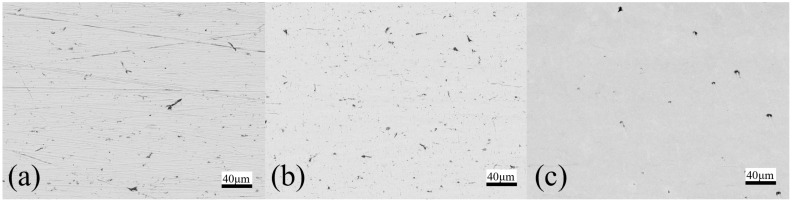
SEM photographs of workpiece (**a**) before polishing (**b**) rough polished (**c**) fine polished.

**Figure 11:**
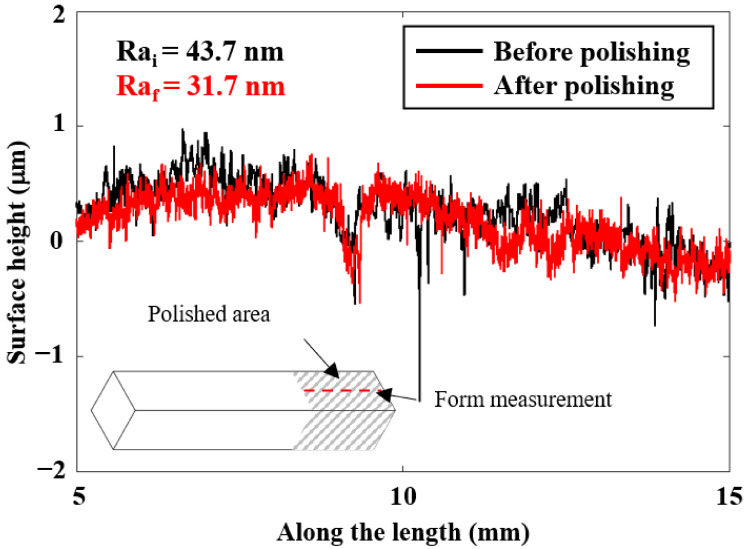
Surface profiles of square bar plane 4.

Figure 8 presents the snapshots of the fine polished surface of a square bar. It can be seen that plane 4 shows a much clearer reflected image than the other three planes, whereas plane 3 shows the poorest. The surface roughness profile of plane 4 of the square bar before and after polishing was shown in Figure 9. It is observed that the rough peaks were largely sheared off after the polishing process as shown in Figure 9a, and the overall surface height deviations were improved. Figure 9b,c shows the 3D contour of surface roughness measured by a Zygo Nexview white light interferometer, and the arithmetical mean height ($S_{a}$) has improved from 146 nm to 26 nm, and the root-mean-square roughness has also reduced from 193 nm to 36 nm, which signifies the polishing performance of the process. Figure 10 shows the surface integrity of the workpiece captured by a Scanning Electron Microscope (Hitachi tabletop microscope-TM3000). Most of the scratches and defects have been removed after rough polishing and the surface was further smoothened after fine polishing. Figure 11 demonstrates the comparison of the surface profile before and after polishing, indicating that the surface form accuracy can be well maintained along the height direction of the square bar after the MAMP process.

### 3.2. Polishing Performance on the Roller Surfaces

**Figure 12:**
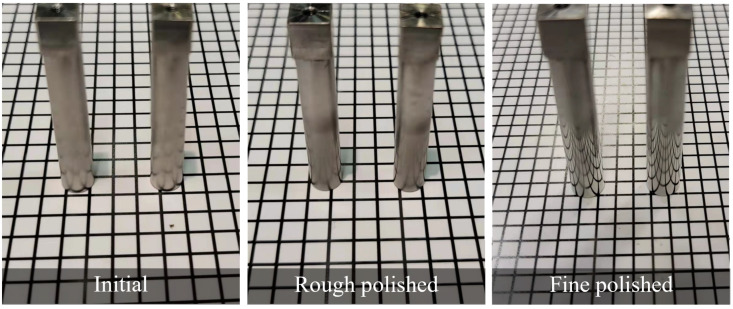
Snapshot of roller workpieces before and after polishing.

**Figure 13:**
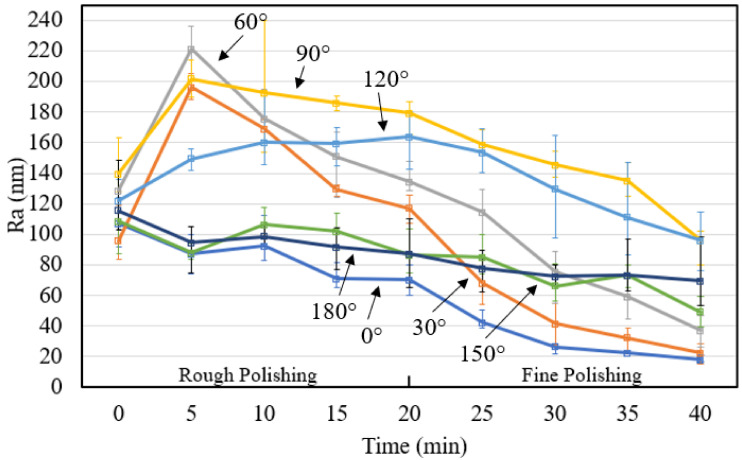
Surface roughness change of roller workpiece at different angles.

Roller type workpieces were polished to examine the effect of the polishing angles. A snapshot of the roller surface before and after polishing was shown in Figure 12, where a smoother surface was obtained after rough polishing, and a shiny and reflective surface can be observed after the fine polishing process. The rough polished and fine polished surface roughness change was shown in Figure 13, and the varied polishing performance can be observed after fine polishing, where 0°, 30° and 60° have experienced a more dramatic fall in surface roughness reaching around 25 nm; 150° and 180° shows a very gradual decrease during both rough and fine polishing; and 90° and 120° received nearly no improvement in the rough polishing process and a slight decrease after the fine polishing process. From the results, under the same impact angle, where 0° corresponds to 180°, a different value was obtained after the whole polishing process; better polishing performance was observed near the external chamber wall and the reason is discussed in the section below.

### 3.3. Simulations

#### 3.3.1. Simulation of the Magnetic Field Distribution

**Figure 14:**
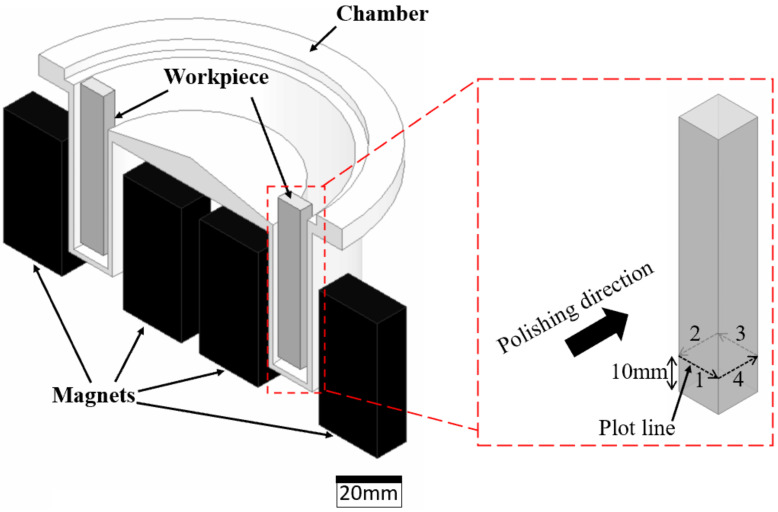
Finite element analysis model.

**Figure 15:**
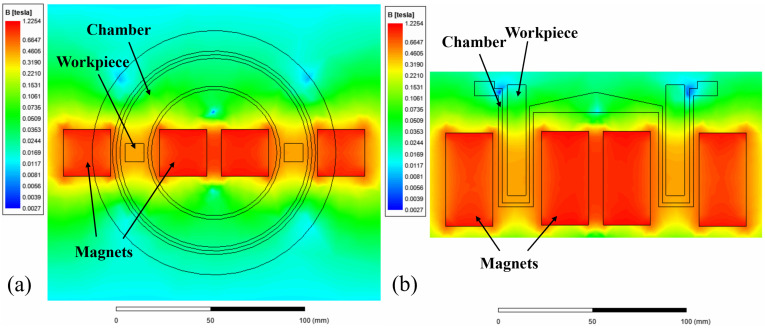
Simulation result of magnetic flux density distribution in (**a**) top view (**b**) front view (sectioned).

**Figure 16:**
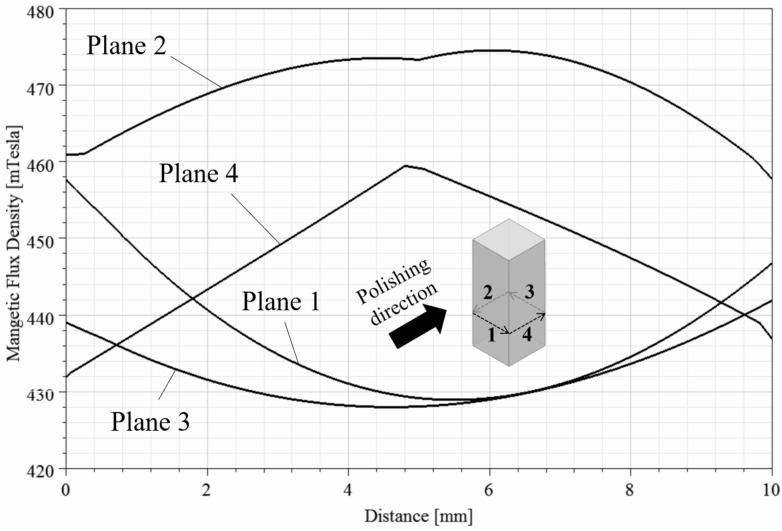
Simulation result of magnetic flux density horizontally along each workpiece plane.

In MFAF, the magnetic force is critical to the polishing performance. Hence, the magnetic flux density distribution in MAMP was modelled based on the finite element method (FEM) to explain the phenomenon of uneven roughness results. A simulation of the magnetic flux density distribution was performed using ANSYS Maxwell. A magnetostatic solver was adopted to solve Maxwell’s equations in a setup as shown in Figure 14. The default boundary condition with 100% padding was applied to ensure sufficient room for fringing, and an automatic adaptive meshing for 10 passes was set. Four non-model lines were drawn on the surface of the four planes of the workpiece to extract the numerical value of the magnetic flux density of the different planes. Figure 15 shows the magnetic flux density distribution of the system, and Figure 16 presents the graphical value of magnetic flux density along the horizontal line of each plane. From Figure 15 and Figure 16, plane 1 and 3 were experiencing a weaker magnetic field of 430 mT compared to 470 mT in plane 2 and 450 mT in plane 4. This is due to the increase of distance between the magnets and the plane, as plane 2 and 4 were closer to the chamber wall and parallel to the magnets surface, thus experiencing a higher magnetic force. Whereas plane 1 and 3 lies in between the two magnets, it experiences magnetic force from both sides of the permanent magnets at the edges but less magnetic forces in the middle of the plane. Theoretically, a stronger magnetic force indicates a tougher magnetic abrasive brush as the bonding in the magnetic abrasive chain is stronger, thus areas near the chamber wall should have a higher material removal rate.

#### 3.3.2. Simulation of Impingement

**Figure 17:**
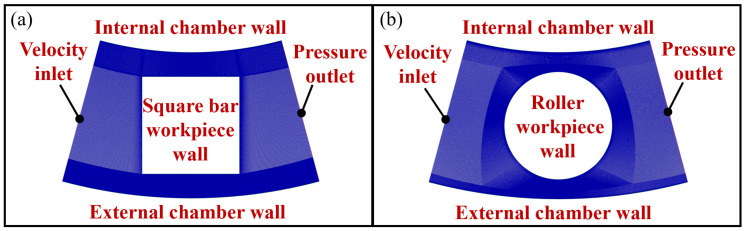
Mesh generation in CFD model simulation of (**a**) square bar (**b**) roller workpiece.

**Figure 18:**
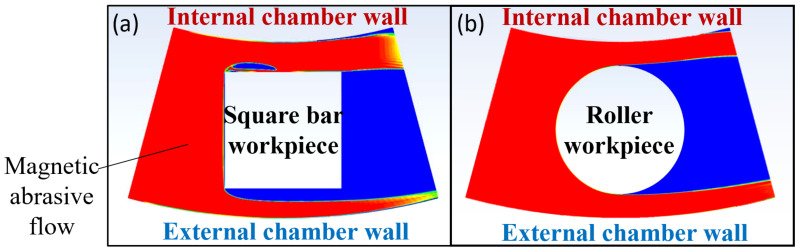
Simulation result of fluid flow in the polishing process of (**a**) square bar (**b**) roller.

In addition to the magnetic field distribution, the movement of the magnetic flow can also be attributed to the difference in polishing performance at different orientations. Hence, the movement of the fluid flow was also modelled by ANSYS Fluent in this study to provide a deeper understanding on the effect of the fluid flow movement when polishing different shapes of the workpiece. The rotational symmetry structure during polishing makes it possible to simplify the simulation down to a 2D axisymmetric problem. The Navier-Stokes’s equation with incompressible form is applied to solve the fluid velocity field. To describe multiphase systems, a simple algorithm and Volume of Fluid (VOF) model were employed to solve the pressure-velocity coupling and model the continuous multiphase, respectively. Considering the effect of turbulence on flow field, the Shear-Stress Transport (SST) was used to express the turbulent fluid flow in the inner region of the boundary layer as well as in the outer part of the boundary layer for a wide range of the Reynolds number. The inlet was defined as the velocity inlet with a velocity of 6.6 m/s, which is calculated based on 1500 revolutions per minute, with a 42 mm distance from the rotational axis; and the outlet was a pressure outlet. Furthermore, the fluid viscosity was set to 0.001 kg/s to simulate the property of polishing slurry. Figure 17 shows the mesh generation methods, and all the boundary layers were refined to guarantee the simulation accuracy. Looking into Figure 18, according to the polishing direction, the abrasive brush flows from the left to the right, first impinges plane 1 in Figure 18a, then separates into two streams and rubs the surface of plane 2 and plane 4, while plane 3 remained uncontacted. As for the roller workpiece as shown in Figure 18b, the abrasive flows along the curvature, so half of the workpiece was polished while the other half at the back was not.

## 4. Discussion

### 4.1. Discussion on the Effect of Surface Orientation

The experimental results show that the MAMP method has different polishing performance at different posture and orientation; better polishing performance can be observed in the surfaces parallel to or slightly inclined to the polishing direction. In the square bar workpiece, plane 2 and 4 shows significant roughness reduction, plane 1 has little improvement and plane 3 has no obvious change. Whereas in the roller workpiece, 0° and 30° impact angles show the lowest roughness after the polishing process. This result can be explained by the varying abrasive brush stiffness and abrasive flow. The stiffness of the abrasive brush is considered to be related to the magnetic pressure which is mainly produced by magnetization. This magnetic pressure expression can be written as follows [40]

(1) $$P_{m}=\frac{B^{2}}{2\mu_{0}}\left(1-\frac{1}{\mu_{r}}\right)$$

where *µ*_0_ is the free space permeability, *µ_r_* is the relative magnetic permeability of the magnetic brush, and *B* is the magnetic flux density on the target surface, which can be obtained from the simulation model of magnetic field distribution. It is known from Equation (1) that the greater the value of *B*, the larger the *P_m_*. According to the magnetic field simulation results, the magnetic flux density near the chamber wall was stronger than that of the middle region by around 40 mT, and magnetic force is found to be proportional to the magnetic field strength [41]. Thus, it can be deduced that a stronger magnetic pressure can be found near the chamber wall (plane 2 and 4) while a lower magnetic pressure was formed at the middle of the tunnel (plane 1) which would lead to a weaker impingement and material removal. Whereas plane 3 was not able to be polished as demonstrated in the CFD simulation shown in Figure 18. From the roller type experiments, a perpendicular impact angle shows poorer improvements, while 0° and 30° polishing angles had achieved the lowest surface roughness of 20 nm, which also conforms to the results of the bar type workpiece. In addition, the perpendicular polishing angle has not allowed effective shearing, as the magnetic abrasives were stopped by the surface when hitting the perpendicular surface, resulting in near zero velocity along the polishing direction that material removal can hardly occur, whereas an angled surface allows the abrasives to slide through and shears off the material from the surface during the impingement, and thus the middle area of the roller workpiece appears to have a higher surface roughness after polishing.

However, although plane 2 and plane 4 of the square bars were both parallel to the polishing direction, and that 0° and 180° of the rollers shall share the same impact angle, the polishing performance of these identical angle pairs did not show a similar efficiency. It was found that lower surface roughness values were recorded in surfaces near the outer circle (external chamber wall), especially during the fine polishing process, as compared to surfaces near the inner circle (internal chamber wall) despite the stronger magnetic force in the inner circle. This might be due to the centrifugal force brought by the rotational movement of the abrasive brush. In the polishing process, magnetic abrasives were attracted to two pairs of magnetic poles, and rotated at a high-speed of 1500 rpm; centrifugal force can be loaded on the magnetic abrasives which tend to be swung outwards, leading to a less concentrated brush in the inner circle of the chamber. The magnetic force *F_m_* on the magnetic particles under the magnetic field can be expressed by [42]:

(2) $$F_{m}=V\cdot X_{m}\cdot H\cdot \nabla H$$

where V is the volume of magnetic particle and $X_{m}$ is the mass susceptibility, *H* is the magnetic field strength and ∇H is the gradient of *H*. From Equation (2), it is known that with a greater volume of magnetic particle (*V*), a larger magnetic force (*F_m_*) can be obtained. Similarly, in fine polishing, the magnetic particles are smaller in size and loosely bonded, and a weaker magnetic force and linkage was experienced as compared to the large magnetic abrasives used in rough polishing. Thus, the weak magnetic force may not withstand the strong centrifugal force experienced by the fine polishing abrasive as reported by Shukla and Pandey [43], abrasives in the inner circle would have been thrown outwards and accumulate on the external wall that area near internal chamber wall cannot be reached by the abrasives, leading to the different polishing pattern between rough and fine polishing. Thus, with the current design of the MAMP system, the target surface should be mounted facing outwards to ensure the best polishing performance.

### 4.2. Discussion on the Effect of Different Region on the Same Surface

**Figure 19:**
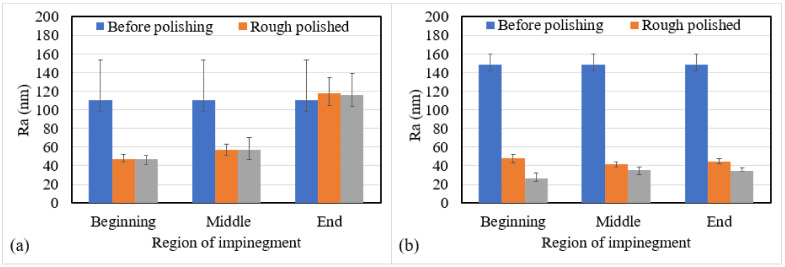
Polishing performance at different regions of impingement in square bar (**a**) plane 2 and (**b**) plane 4.

Looking into the performance in the parallel direction of the square bar workpiece, the results show that regions at the beginning of impingement were better polished in both plane 2 and 4, while plane 4 had a larger variation of roughness among the three regions after fine polishing. The fluid simulation also conforms with the polishing results that only plane 1, 2 and 4 were able to be reached, with fluid being slightly blocked apart from the surfaces of both plane 2 and plane 4. Figure 19 has summarized the polishing performance at different regions of impingement in plane 2 and 4, while plane 2 region C and plane 4 region A belongs to the beginning of impingement while plane 2 region A and plane 4 region C belongs to the end of the impingement. Generally, only regions at the beginning and middle can be significantly polished. Magnetic abrasives may be retained at the highest point of the workpiece (at the middle of region B), so that the sliding of the abrasive cannot continue on regions at the end of the impingement. With the magnetic abrasive being blocked due to the surface shape, the high flow-speed magnetic abrasive brush might not have allowed enough time for the abrasives to re-link themselves under the magnetic force and thus cannot completely conform to the surface, leading to a varying polishing performance. To overcome this problem, a larger gap distance between workpiece surface and the chamber wall is believed to be able to allow smoother pass through of the stiffened brush, and thus a more complete contact. Moreover, keeping the target surface at an inclined angle against the polishing direction is important to allow efficient shearing.

### 4.3. Discussion on the Edge-Rounding Effect of Square Bar Surface

**Figure 20:**
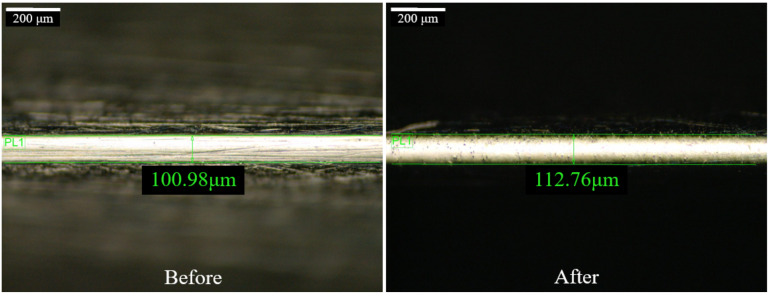
Edge width of square bar workpiece before and after polishing.

After a 20-min rough polishing and 20-min fine polishing, the square bar component has a great surface roughness improvement on plane 4, especially at the beginning area of impingement. Figure 20 shows the edge width of the square bar before and after polishing. It can be observed that the edge width has a slight increase from approximately 100 μm to 110 μm after polishing, and the sharp and clear edge (white area) has diminished slightly, symbolizing a small round edge despite the similar width of the white area. The edge rounding effect is mainly induced by the rough polishing process as the abrasive particle size was larger, however, since the rough polishing process was not performed for a long period, the edge rounding effect was insignificant in this study. In addition, the edge rounding effect during rough polishing can be minimized by controlling the polishing time and polishing angle, whereas in fine polishing, edge-rounding effect was not found [38].

### 4.4. Discussion on the Methods to Improve the Polishing Uniformity

From the experimental results, it can be deduced that the target surface should be facing outwards when polishing a non-revolving surface. Moreover, an effective polishing angle should be determined to allow uniform material removal. For a revolving surface (i.e., roller) that requires polishing of the entire surface, the extra rotation of the workpiece can be added to ensure uniform polishing of the whole surface. Driving the magnetic brush to run both in a clockwise and anti-clockwise direction should also be helpful to obtain uniform polishing of the whole surface. Another possible approach might be controlling the workpiece to self-rotate or performing overlapping polishing by adding a motor to match the polishing direction and impact angle. As it was found that an area with 0–30° impact angle achieves better performance, therefore by rotating the component and changing the orientation of the surface to the effective polishing angle repetitively and constantly, the material removal uniformity can be improved.

## 5. Conclusions

In this study, experimental investigation and simulation on the effect of surface shapes and orientations in the MAMP process was conducted on the square bar and roller. The obtained conclusions are as follows:

(1) Surfaces near the chamber wall experience higher magnetic strength, a stiffer magnetic brush is formed and thus generally performs better, and the target surface should be mounted facing outwards.
(2) Regions at the beginning of impingement were polished better, as the abrasive brush was either obstructed or not conforming to the regions behind it due to the workpiece shape and high rotational speed; polishing angle adjustment will be needed to eliminate the limitations.
(3) Both types of workpieces have partially achieved a final surface roughness of Ra = 20 nm after fine polishing.
(4) Further investigation is still needed to study the polishing mechanism and improve the polishing uniformity.

## Figures and Tables

**Table 1:** Polishing parameters in experiments.

Parameters	Values
Rotational speed	1500 rpm
Polishing abrasive	Rough polishing 500~1000 µm Al_2_O_3_ sintered magnetic abrasives Fine polishing ~2 µm CIP (80 wt.%) + 150 nm Al_2_O_3_ (20 wt.%)
Polishing time	20 min
Workpiece	Square bar (10 × 10 × 60 mm); SS304 Roller (Ø10 × 60 mm); SS304
Magnets	N52 Neodymium permanent magnets, 25.4 × 25.4 × 50.8 mm;
